# Bipolar disorders and suicide: stumbling twice with the same stone?

**DOI:** 10.1192/j.eurpsy.2022.579

**Published:** 2022-09-01

**Authors:** M. Sagué-Vilavella, G. Fico, G. Anmella, A. Giménez-Palomo, M. Gómez-Ramiro, M. Pons Cabrera, S. Madero, E. Vieta, A. Murru

**Affiliations:** 1 Hospital Clínic de Barcelona, Department Of Psychiatry And Psychology, Barcelona, Spain; 2 Complejo Hospitalario Universitario de Pontevedra. SERGAS., Department Of Psychiatry, Pontevedra, Spain

**Keywords:** Suicide, Lithium, bipolar disorders

## Abstract

**Introduction:**

Suicide is the most terrible outcome of bipolar disorders (BD). It impacts families and healthcare professionals deeply. Family history of suicide (FHS) is one of its main risk factors, whereas lithium treatment and absence of substance use disorders (SUD) are two of its few modifiable protective factors.

**Objectives:**

To explore the relationship between FHS and clinical characteristics in BD. We hypothesized that FHS would be associated with less SUD, higher rates of lithium treatment and shorter duration of untreated illness (DUI).

**Methods:**

Cross-sectional analysis of subjects with BD followed-up in a specialised outpatient unit (Barcelona, October’08-March’18). We described data with measures of frequency, central tendency and dispersion, and we used χ², Fisher’s test and t-tests for comparisons.

**Results:**

The sample consisted of 83 subjects, 56.6% males, mean age 41.9 years (SD 12.7). 74.7% (n=62) had a diagnosis of BD-I and 25.3% (n=21) of BD-II. 11 subjects (13.3%) had FHS. Those with FHS did not show significant differences in sociodemographic data, DUI (58.5+/-60.4 vs 38.19+/-84.9 months, p=0.341), lithium use (72.7% vs 73.6%, p=0.95) or SUD (27.3% vs 23.6%, p=0.79). There were differences in terms of lifetime suicide attempts (54.5% vs 20.8%, p=0.026), family history of mental disorders (100% vs 69.4%, n=0.032).

**Conclusions:**

Contrary to our hypothesis, FHS was not associated with the modifiable protective factors against suicide (namely, less SUD and more lithium prescription). Similarly, we did not find an association with earlier access to mental health services at symptom onset (DUP as proxy). Therefore, our results suggest FHS does not modify attitudes towards prevention.

**Disclosure:**

No significant relationships.

